# Restrictive Deterrence in Drug Offenses: A Systematic Review and Meta-Synthesis of Mixed Studies

**DOI:** 10.3389/fpsyg.2021.727142

**Published:** 2021-08-25

**Authors:** Xin Guan, T. Wing Lo

**Affiliations:** Department of Social and Behavioral Sciences, City University of Hong Kong, Hong Kong, China

**Keywords:** drug offense, restrictive deterrence, certainty and severity of punishment, meta-synthesis of mixed studies, punishment avoidance

## Abstract

Deterrence by punishment aims to prevent a crime; however, it is not always successful. Restrictive deterrence explains the continuous criminal activities that occur despite deterrence; offenders enact various strategies to avoid detection, which is more typical among drug offenders given that they have a high frequency of offending and exposure to punishment. This systematic review provides an in-depth understanding of restrictive deterrence of drug offenders. Two prominent themes, “restrictive deterrence strategy” and “deterrability and restrictive deterrence,” depict drug offenders' restrictive deterrence and effectively fit within the certainty–severity framework of punishment. Future studies should investigate restrictive deterrence strategies in the after-arrest context, the facilitative effect of perception of risk on strategy development, and facilitators or inhibitors affecting the diffusion of restrictive deterrence strategies.

## Introduction

For decades, researchers, and theorists in criminology have investigated punishment and its deterrent effect. A large portion of deterrence research has focused on how punishment exerts an influence on people's determination to engage in or refrain from illegal behavior. Two elements of punishment, certainty, and severity, are the most commonly cited and explored, and are considered to be influential factors in motivating people to avoid committing crimes. Although punishment aims to deter people from crime altogether (absolute deterrence), it has a chance of encouraging people to commit crimes in insidious ways, which echoes the concept of restrictive deterrence (partial deterrence) (Gibbs, 1975, p. 33).

The distinction between absolute and restrictive deterrence is the extent to which people adjust their criminal behavior in reaction to risks. As Gibbs (1975, p. 32) defined, absolute deterrence denotes “an individual has refrained throughout life from a particular type of criminal act because in whole or in part he or she perceived some risk of someone suffering a punishment as a response to the crime.” Restrictive deterrence denotes “the curtailment of a certain type of criminal activity by an individual during some period because in whole or in part the curtailment is perceived by the individual as reducing the risk that someone will be punished as a response to the activity” (Gibbs, 1975, p. 33). It can be derived from two definitions that some persons may stop committing crimes to lessen their likelihood of punishment, while others may only curtail the frequency of crime. Beyond the magnitude of behavioral change, the two kinds of deterrence apply to different types of offenders. Absolute deterrence pertains to the people who refrain from participating in crime from a time onwards, regardless of their previous crime involvement. However, restrictive deterrence is only applicable to those who have committed a particular crime at least once.

Jacobs (1996a) expanded Gibbs's definition of restrictive deterrence by classifying it into two distinct types: probabilistic and particularistic restrictive deterrence. The former corresponds to the definition proposed by Gibbs (1975, p. 33), which emphasizes the reduction of crime frequency. The latter refers to the “skills for evasion” (Jacobs, 1996a, p. 425), implying that offenders develop various situational measures, namely restrictive deterrence strategies, to carry out an offense more likely to go undetected. For example, an offender committing street crimes takes advantage of everyday social activity to disguise the act of committing a crime (e.g., shaking hands with another using complex street handshake etiquette while holding an illegal substance in his hands and exchanging it with his partner). An offender commits offenses of lesser severity than the one anticipated because he believes that there will be less penalty for a less serious crime (e.g., an offender only sells cannabis rather than heroin). Both of these are typical restrictive deterrence strategies among drug offenders.

Drug offenders, referring to those who use, deal/traffic, or cultivate/manufacture illegal drugs, are of particular relevance in the theoretical development of restrictive deterrence. Since Gibbs (1975, p. 33) introduced the concept of restrictive deterrence, it has been substantively explored on samples of drug offenders (Jacobs, 1993, 1996a,b). Originally based on research concerning drug offenders, restrictive deterrence was also gradually extended to a broader range of criminals, such as auto thieves, sex workers, sexual offenders, and computer hackers, among others (Jacobs and Miller, 1998; Cherbonneau and Copes, 2006; Beauregard and Bouchard, 2010; Gallupe et al., 2011; Jacobs and Cherbonneau, 2014; Maimon et al., 2014; Wilson et al., 2015).

Likewise, restrictive deterrence is of particular relevance in shaping the character of drug offenders. First, the high recidivism levels of drug offenders (Harrison, 2001) and their involvement in multiple crimes (Casey, 2015) suggest that they are among the most judicially entrenched offenders. The high recidivism rate of drug offenders may be partly because restrictive deterrence strategies facilitate them to avoid arrest and thus build a criminal career. Second, the restrictive deterrent effect is more potent for drug offenders than liquor drinkers, petty thieves, or vandalizers (Paternoster, 1989; Eck and Wartell, 1998). The high risk-responsiveness of drug offenders may be partly because restrictive deterrence contributes to converting drug offenders' risk perception into action against risk rather than just quitting from crime or ignoring the risk.

Drug offenders evolve strategies to counteract the threats of punishment, and punishment threats are developed in return to discourage offenders more efficiently; such progress repeatedly continues and becomes an inevitable cycle (Ryan, 1994). Drug offenders have shown their adaptiveness to cope and innovate ways to commit crimes. Consequently, practitioners of criminal justice and scholars need to thoroughly grasp restrictive deterrence to better respond to newly-developed patterns in drug offender behavior.

The bulk of this systematic review examined restrictive deterrence of drug offenders, including the concrete strategy and possible prerequisites for strategy use. The current systematic review synthesizes 34 quantitative, qualitative, and mixed-method studies that focused on restrictive deterrence of drug offenders, and analyses findings based on a certainty–severity framework of punishment, providing an explicit picture and revealing the understudied field of restrictive deterrence of drug offenders.

To complete the synthesis and interpretation, we introduce a certainty–severity framework of punishment (**Figure 2**). Punishment is a system of conditional probabilities (Nagin, 2013), including multiple probabilistic events between arrest and final sentencing. As the sequence of probabilistic events evolves, the certainty and severity of the punishment also undergoes an increase. The certainty and severity of punishment serve as deterrents (Piliavin et al., 1986; Williams and Hawkins, 1989), where the certainty of punishment is viewed as the most influential (DeJong, 1997; Pogarsky, 2002; Nagin and Pogarsky, 2003) and the severity of punishment only produces a modest effect (Pogarsky and Piquero, 2003; Apel, 2013). The framework based on these two elements of punishment assists in the better visualizing of restrictive deterrence of drug offenders.

In the certainty–severity framework of punishment, the x-axis represents the certainty of punishment and the y-axis represents the severity of punishment. Along the x-axis and y-axis, different restrictive deterrence strategies are presented, and in the middle of this coordinate system factors that influence the strategy implementation are listed. The clustered themes scatter along axes and the coordinate system, explaining how drug offenders implement strategies to “move” to the origin place (0,0) representing successfully avoiding detection, and exploring the potential prerequisite for strategy implementation.

## Materials and Methods

### Meta-Synthesis of Mixed Studies

In the present study, meta-synthesis of mixed studies is adopted. Meta-synthesis is an analytical technique used to combine and compare the outcomes or metaphors of various qualitative studies to create interpretations, ground narratives, or theories (Sandelowski et al., 1997; Beck, 2002). Meta-synthesis expands the qualitative results by analyzing the distinctiveness of a study as a through and interpretive whole as opposed to a meta-analysis, which transforms quantitative research into averages (Clemmens, 2003). Though frequently focused on qualitative research, it can also be used to integrate qualitative, quantitative, and mixed-method studies to provide a more holistic view of the problem than could be obtained from a one study approach (Panda et al., 2018). A meta-synthesis is still not commonly used and is a relatively new method in criminology. Nevertheless, it is a worthy instrument to promote gap-finding. Therefore, the meta-synthesis of mixed studies might be an effective way to achieve a thorough analysis of restrictive deterrence of drug offenders.

### Procedure of Meta-Synthesis of Mixed Studies

The review consists of four successive phases: data selection, data extraction, theme identification, and finding synthesis.

#### Data Selection

**Figure 1:**
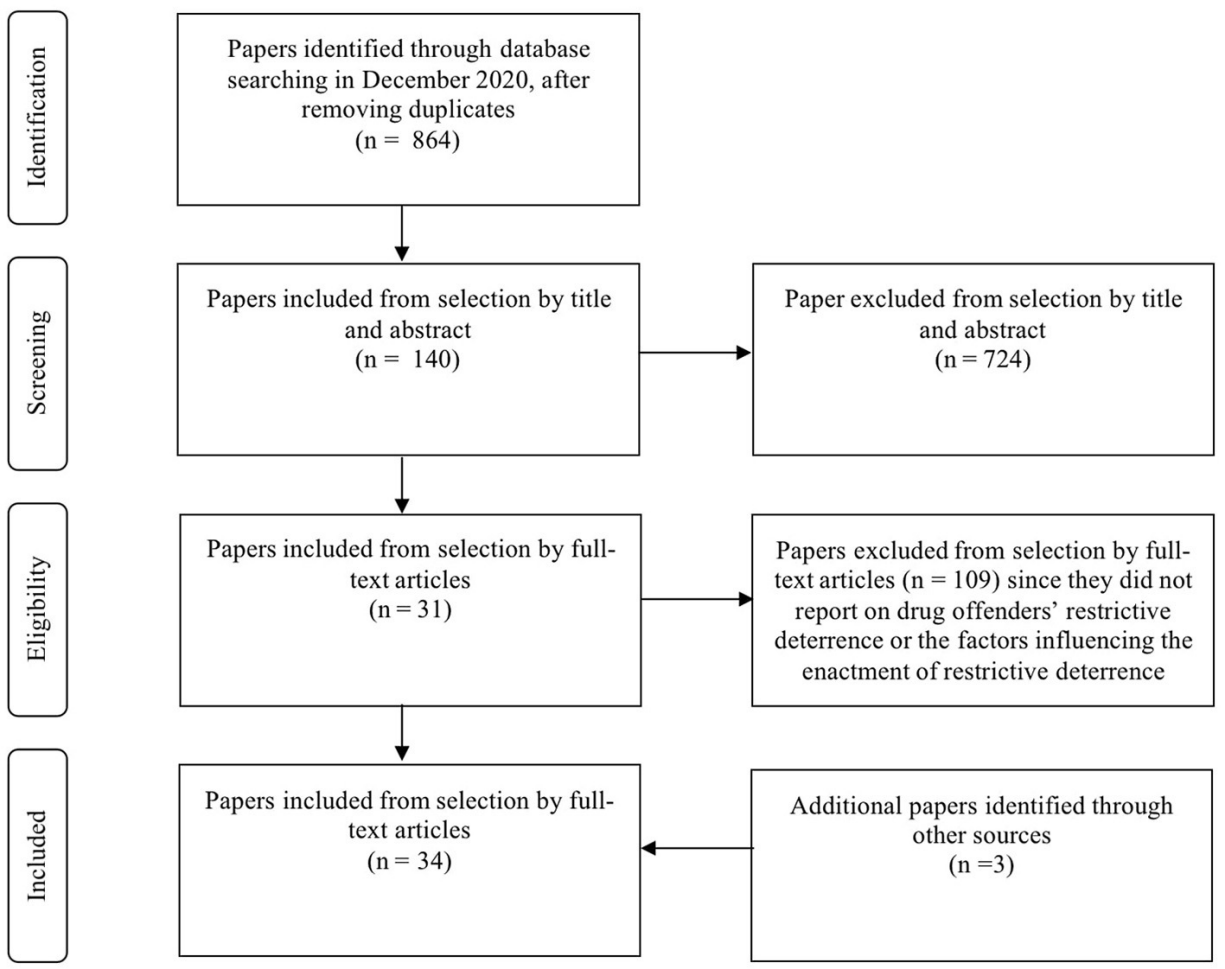
PRISMA flow chart presenting search results.

The review complies with the guideline of Preferred Reporting Items for Systematic Reviews and Meta-Analyses (PRISMA) (Moher et al., 2009) (Figure 1).

The HeinOnline, Social Science Database, Sociological Abstracts, Scopus, SAGE, JSTOR, PsycINFO, and Web of Science research databases were searched in December 2020. Furthermore, the SPIDER (Sample, Phenomenon of interest, Design, Evaluation, Research type) approach (Cooke et al., 2012) was used to decompose the targets and reinforce the search strategy (see Appendix 1).

Data screening was proceeded in three steps. The first step was de-duplication. The second step was screening with titles. Researchers followed the idea of the SPIDER approach, leaving those studies with titles containing terms such as “drug” and its derivatives, “deterrence” and its cognates or “avoid” and its cognates. The third step was screening with abstracts. As the drug-related studies have a broad perspective, the researcher only selected studies with their abstract indicating how drug offenders commit crimes or the factors influencing the ways they commit crimes, and excluded those studies that focused on the subjects of law enforcement, victims of drug crime or other subjects involved in drug crime.

After the data screening, the Mixed Methods Appraisal Tool (MMAT) version 2018 was used to evaluate the methodological quality of the included studies. The MMAT contains 27 methodological quality criteria for appraising qualitative, quantitative and mixed-methods studies. Each criterion in the tool would be labeled with one asterisk if a requirement was met and would be labeled without asterisk if a requirement was not met or “cannot tell.” There are two comprehensive screening questions for all types of studies, namely “Are there clear research questions?” and “Do the collected data allow to address the research questions?” A further appraisal may not be feasible when the answer is “no” or “cannot tell” to one or both screening questions. Of the remaining 25 questions, 5 of them are expressly set up for appraising the qualitative study, 15 of them for quantitative study (as the tool divides quantitative study into three types, including quantitative randomized controlled trials, quantitative non-randomized and quantitative descriptive), and 5 of them for mixed-methods study. Accordingly, for each study, it will be labeled with 5 asterisks, or scored 100%, if it meets all criteria for the type of study. By analogy, a study that meets 4 criteria will be labeled with 4 asterisks (or scored 80%). The MMAT does not have a specific standard cut-off value. However, two categories (low and high) or three categories (low, medium and high) can be adopted. The crucial aspect is to carefully utilize the results of the appraisal in the review. One author of the present study analyzed the methodological quality of each included study and verified its final score. Studies with at least 3 asterisks (or scored 60%) were kept.

#### Data Extraction

A data extraction table was developed to report a full description of each included study, including its purpose, sample size, design, participants, research setting, and the drug crime type reported by the author.

#### Theme Identification

Themes were extracted and grouped from individual studies into wider themes and subthemes before being synthesized. A thematic analysis and meta-synthesis were performed rather than a meta-analysis since meta-analyses are not feasible when there is considerable heterogeneity among qualitative studies.

#### Synthesis Identification

Based on the search strategy, 1,237 individual titles were retrieved and 864 studies remained after removing any identified duplicates (*n* = 373) (Table 1). A total of 724 studies were excluded after reviewing their “titles” and “abstracts.” Hence, 140 studies remained for a full-text review by the author, and 109 of these were excluded since they failed to report drug offenders' restrictive deterrence or the factors influencing restrictive deterrence. Meanwhile, an additional three papers identified through other sources were added (Figure 1). Finally, 34 included papers were reviewed for quality appraisal. All of these papers scored as moderate (score of 60 to 80%) (*n* = 10) or high quality (score of 100%) (*n* = 24).

**Table 1:** Results of search strategy for each database.

**Database**	**Year**	**Results**
Heinonline	1975–2020	618
Social science database	1975–2020	172
Sociological abstracts	1975–2020	160
Scopus	1975–2020	159
SAGE	1975–2020	78
JSTOR	1975–2020	24
PsycINFO	1975–2020	14
Web of science	1975–2020	12
**Total**		**1,237**

The 34 studies were published from 1984 to 2019: 18 studies focused mainly on factors that affect the action of restrictive deterrence; 15 of them depicted restrictive deterrence strategies; and only one literature review discussed restrictive deterrence with respect to multiple crimes. Studies were carried out mostly in the United States and other Western countries. Nine studies used a quantitative design (surveys/questionnaires/systematic observations), whilst 22 used qualitative designs (individual or focus group interviews), and three were designed using mixed methods (interviews and surveys/systematic observation).

**Figure 2:**
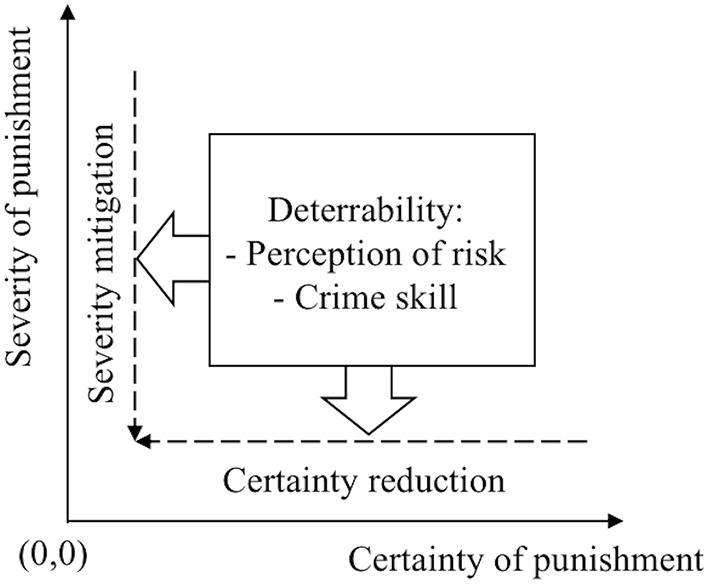
Theoretical framework.

Based on the systematic review and meta-synthesis of mixed studies, the characteristics of the included studies were summarized in Appendix 2 and the most noticeable themes and subthemes in Table 2. Specifically, three main areas of concern were identified: restrictive deterrence strategies, the contingency of restrictive deterrence, and the iteration of restrictive deterrence. These themes fit within the certainty–severity framework of punishment (Figure 2). Our examination of the constitution of the differences within the themes is reflected along these axes. In the subsequent section, a series of inferences and generalizations about restrictive deterrence of drug offenders and any uncharted areas are explored.

**Table 2:** Restrictive deterrence domains and strategy used by drug offenders.

**Theme**	**Subtheme**	**Detail**
Restrictive deterrence strategy	1. Certainty reduction	a. Camouflage in front stage (Jacobs, 1996b; Jacobs and Miller, 1998)
		b. Pick safe time and position (Jacobs and Miller, 1998; VanNostrand and Tewksbury, 1999)
		c. Counter-reconnaissance (Jacobs, 1993, 1996a; Jacques and Reynald, 2012)
	2. Severity mitigation	a. Choose less severe activity (Knowles, 1999; Fleetwood, 2014)
		b. Pass risk (Knowles, 1999; Piza and Sytsma, 2016)
		c. Stash product (Jacobs, 1996b; Jacobs and Miller, 1998; Moloney et al., 2015)
		d. Cooperate with police (Cross, 2000; Dickinson and Wright, 2015)
Deterrability and restrictive deterrence	1. Perception of risk	a. Individual characteristic
		b. Crime milieu characteristic
	2. Crime skill	a. Self-reflection
		b. Collective wisdom

## Results

### Restrictive Deterrence Strategy

Offenders are risk-respondents rather than risk-takers (Jacobs and Cherbonneau, 2014). In other words, offenders adopt various strategies to alter the risk environment in which they are placed. Punishment is one of the most significant risks associated with offenders. It can mainly be divided into two types. One is formal punishment (legal punishment), such as arrest/apprehension. Another is informal punishment (e.g., moral sanction) (Jacques and Allen, 2014), such as stigmatization/labeling. Informal punishment, at some level, can be seen as a subsequent punishment triggered by formal punishment (Nagin, 1998). Therefore, all allusions to punishment in the following indicate formal punishment.

Many terms such as detection, arrest, apprehension, conviction, prosecution, and sanction have been used to refer to punishment or a part of punishment. There is an obvious need for clear and coherent definitions of punishment and/or its associations in light of the expansion of literature. As Nagin (2013) noted, punishment is more accurately characterized as a system of conditional probabilities. There are multiple probabilistic events between detection and final sentencing, such as from arrest to detention, to prosecution, to conviction, and to sentencing. In this sequence of conditional probabilities, punishment is shown as a process of severity in legal or judicial responses.

We extended this concept of punishment to the restrictive deterrence strategies of drug offenders. Drug offenders adopt various strategies to minimize their odds of arrest. These strategies help avoid one kind of adverse event. They also impact the probability of the occurrence of the subsequent event and affect the final sentencing. Hence, in what follows, the restrictive deterrence strategy used by drug offenders, whether to avoid arrest or to reduce the length of a sentence, can be understood as an attempt to avoid the punishment.

The commonly used classification of restrictive deterrence strategy is contended by Jacobs (1996b), including probabilistic strategies and particularistic strategies. However, the current study adopts Moeller et al.'s (2016) classification of restrictive deterrence strategies because it is in line with the certainty–severity framework of punishment. Hence, restrictive deterrence strategies are divided into certainty reduction strategies and severity mitigation strategies. The former corresponds to criminal thinking about “what should I do to commit a crime while keeping myself from arrest,” and the latter corresponds to “what should I prepare to do if I am arrested?”

#### Certainty Reduction: “I Need a Plan”

Strategies of certainty reduction are designed to allow drug offenders to remain “invisible” to police when a crime occurs. Drug offenders disguise themselves under the cloak of legal activities, keeping a low-profile, choosing a less-risky time and area, and discreetly uncovering their adversary's invasion. In such a way, they create an illusion of being a law-abiding person.

##### Camouflage

Drug offenders camouflage their drug offending in two ways: integrating crimes into existing legal daily routines or producing staged performances to disguise offenses and allow them to evade police attention continually. Jacobs and Miller (1998) noted that drug dealers make good use of gender advantages to blend into their environments. Female drug dealers take their children to transactions and project a self-image that does not use dramatic clothes and accessories to reduce police suspicion (Jacobs and Miller, 1998; Carbone-Lopez, 2015; Moloney et al., 2015). Some drug dealers engage in legal occupations or a legitimate business because these can generate unpredictable street activity routines which can reduce law enforcement (VanNostrand and Tewksbury, 1999; Fader, 2016b, 2019). Some people who sell drugs need to cooperate with others. They have to set up a flawless “stage performance” by preparing a set of props, feasible locations, helpmates, and even specialized words, the so-called “transactional mediation” contended by Jacobs (1996b). This requires that buyers be regular customers who commonly understand “the same kind of action” (Schutz, 1972, p. 155). Jacobs (1996b) identified three ways of using transaction mediation: flash decoys, moving screens, and sleight of hand. Flash decoys refers to finishing the drug deal in automobiles, camouflaging the whole process as a kind of favor to hitchhikers. Moving screens refers to rehearsing the movements between participants to speed up the transaction and smoothness of dealing. Sleight of hand refers to using normal hand gestures, including slaps and hugs, to finish the final step of drug delivery.

##### Picking a Safe Time and Position

Deciding when and where to commit the crime is also a significant part of certainty reduction. Drug offenders only commit crimes in locations they consider safe (Carbone-Lopez, 2015; Olaghere and Lum, 2018). A transaction can be arranged at local entertainment facilities, such as restaurants or markets where participants can eat and shop, appearing no different from others to evade intense surveillance (Jacobs and Miller, 1998). For others, selling from home is an ideal option. This decreases the sense of insecurity from being on the street (Moloney et al., 2015). Female drug dealers usually invite friends for after-parties in which drug dealing has been established (Fleetwood, 2014). Along with trading in public areas, some drug trade occurs in secluded lots in which drug dealers can easily perceive risk. Drug dealers guide buyers to walk into a covert space in an apartment while partners watch every move of the buyers if things “go down” (Jacobs, 1996b). Drug cultivators who operate in less visible areas (e.g., stores, hotels) are also more likely to avoid detection (Gallupe et al., 2011). By manipulating their trading location, dealers can squeeze in additional moments to “launder” the illegal income (Jacobs, 1996b). In addition to picking a safe place, timing matters. It is easy to be exposed if selling occurs at an unusual time in the day. Female drug dealers set up an unbreakable principle about opening hours and do not respond to any demands outside the pre-set time (Jacobs and Miller, 1998). Sometimes dealers might suddenly change the location. Drug offenders select areas and times based on police patrol intensity, often diverting to other areas if police patrol is increased in a given region (VanNostrand and Tewksbury, 1999).

##### Counter-Reconnaissance

Drug dealers generally deal with regular customers; however, it is almost inevitable that many of them have connections with unfamiliar, new buyers. Undercover police usually utilize such trading opportunities and operate buy–bust approaches. Thus, offenders develop strategies to identify the presence of police without getting arrested. First of all, they avoid dealing with immature people who could be under intensive police surveillance. Maturity can be judged by whether an individual engages in overly risky behavior, such as an overdose of drugs, or purely based on age stereotypes (Jacques and Allen, 2014). They can also tell police from the real drug customers by their image, in addition to verbal and physical clues, or perhaps test potential buyers in a number of different ways (Jacobs, 1993, 1996a; Johnson and Natarajan, 1995; VanNostrand and Tewksbury, 1999; Jacques and Reynald, 2012). They scan the counterparts repeatedly and collectively to ensure their real identity. To decide if something seems unusual or potentially dangerous, dealers must identify their turf, practicing what they call a “peep game” (Jacobs, 1996b), such as using a foreign language to separate real drug buyers from potential undercover police (Knowles, 1999). Once they identify an undercover police officer, drug dealers withhold exchanges (Jacques and Allen, 2014).

#### Severity Mitigation: “If I Were Arrested”

Compared to fruitful observations about certainty reduction strategies, severity mitigation is understudied (Moeller et al., 2016). One possibility is that severe punishments without certain odds of arrest may have little effect on individual behavior (Carbone-Lopez, 2015). However, many strategies of drug offenders are to prepare for future arrest. Frequently used strategies for severity mitigation include engaging in less severe activity, passing the risk on to associates, stashing stock in converted places, and cooperating with police. In doing so, they believe the probability of a guilty conviction or the severity of their punishment could be decreased if they were prosecuted.

##### Choosing a Less Severe Activity

Choosing a less severe activity is the easiest way to mitigate severity. Some drug runners do not become a dealer because of the fear of hard prison time (Knowles, 1999). Many drug dealers only sell less toxic drugs, such as marijuana rather than crack, because selling crack is regarded as a more severe crime (Fleetwood, 2014; Moloney et al., 2015). Drug cultivators who worry about manufacturing charges perceive cooking and purchasing as precursors that could bring about more severe penalties (Carbone-Lopez, 2015). The drug producers who face manufacturing charges also have several strategies for getting around legislative restrictions to mitigate charges with more severity, such as replacing purchasing precursors with production (Vidal and Décary-Hétu, 2018).

##### Passing on Risks

The passing on of risks is a method of diverting dangers to lower power gang members. The use of selling partners can be a defense against severe charges (Piza and Sytsma, 2016). There are multiple roles in a drug dealing group, with some members charged with the duty to check and receive money and others merely being responsible for drug delivery (Johnson and Natarajan, 1995; VanNostrand and Tewksbury, 1999). In contrast, the “big boss,” who is the actual owner of both the drugs and money, never shows up in the police observation; thus, they could reduce criminal culpability. Lower-level distributors are often hired for the riskiest work (Johnson and Natarajan, 1995; Jacobs and Miller, 1998). Drug runners enable true dealers to be shielded from potential police surveillance or detention, which helps them fully escape the criminal justice system (Knowles, 1999).

##### Stashing Products

Unlike high-level drug dealers who have many helpmates, street drug dealers have to hide drugs in safe locations by themselves to minimize the potential accusation of drug trafficking, which is more severe than drug possession. A frequently used tactic is to hide the majority of their stock and only keep a small number of drugs to be sold quickly (Johnson and Natarajan, 1995). They usually hide the majority of the drugs in caps, under bottles, in newspaper stands, on the ground, or in paper bags that have been placed at a particular angle (Jacobs, 1996b). Women innovate the concealment in their homes, such as a stash inside the hollow shaft of a curtain rod or a box under the carpet over which the pet dog sleeps (Jacobs and Miller, 1998). When carrying the drug, they have to practice drug-handling techniques to avoid scrutiny when they encounter police. Due to the legal constraints stating that police cannot ask suspects to strip, this leaves room to hide drugs within clothes. Drugs are tightly packaged in plastic wrap which can be placed in the hand and mouth without notice or can be swallowed if risk is perceived (Jacobs, 1996b). In drug dealing, women's bodies are viewed as an advantage since they possess “more hiding spots” (Moloney et al., 2015). An on-person or off-person stash is also dependent on settings. In commercial areas with increased foot traffic, an on-person stash is deemed safer than an off-person one (Piza and Sytsma, 2016).

##### Cooperating With Police

To cooperate with the police is to admit drug use if approached by the police (Ribeiro et al., 2010). Fooling the police might lead to violent conflict and even a more severe sentence. Passive cooperation with police means that offenders are “turned” by the police to seek the possibility of a less severe punishment. Such a strategy could be inferred from certain studies. Some sellers have emphasized that they should be careful when dealing with dealers that have recently been charged with a large number of drugs and released soon after because they are more likely to be a decoy under the instruction of the police or an informant (Dickinson and Wright, 2015). Further, the informant may only be charged and convicted as a low-level drug employee (Cross, 2000). In Johnson and Natarajan (1995), a high-level drug dealer recalled that his first jail experience was due to being set up by a drug user.

### Deterrability and Restrictive Deterrence

The aim of restrictive deterrence strategies is to reduce the risk of punishment, or reduce the offenders' perceived risk. This implicitly presupposes that offenders have the ability to perceive and calculate risk. Jacobs (2010) used deterrability to highlight such an ability, explicitly referring to the “offender's capacity and/or willingness to perform risk calculation.” Deterrability is crucial in understanding restrictive deterrence strategies because it supports the idea that such strategies are not arbitrary and thoughtless. Instead, it can be seen as the prerequisite of drug offenders' use of restrictive deterrence strategies.

Jacobs (2010) suggested that deterrability should be measured by risk sensitivity. The current systematic review follows this line of thought and divides drug offenders' deterrability into two parts related to risk sensitivity in criminology, including the perception of risk (Roche et al., 2020) and crime skill (Casey, 2015).

#### Perception of Risk

A growing body of research has highlighted the importance of risk perception in the decision making of offenders (Cherbonneau and Copes, 2006; Beauregard and Bouchard, 2010; Gallupe et al., 2011; Jacobs and Cherbonneau, 2014, 2016, 2017; Maimon et al., 2014; Wilson et al., 2015; Moeller et al., 2016). Perception of risk (subjective risk of punishment) is an extension of actual risk (objective risk of punishment). First, perception of risk is a vital gateway to connect risk and subsequent behavior (Decker et al., 1993; Pogarsky et al., 2004; Paternoster, 2010). Researchers often explain criminal behavior and the vast majority of human behavior by assuming a reality-perception correspondence. Restrictive deterrence does not require a perfect correspondence between the real and the perceived risk. Still, some correspondence (a net positive effect) is necessary if it is to serve as an explanation or a predictor. Second, perception of risk is the individualization of the actual risk for a group of offenders. An example might clarify this. A drug offender lives in a city where 5% of drug offenders are punished each year. This rate of punishment is the average actual risk for both him and the group. However, he is neither a drug lord nor a drug addict who feeds on trafficking but a regular company employee who traffics drugs for subsidizing the household. He is not a gang member and traffics only small amounts of cannabis rather than cocaine. These factors may have reduced his perceived risk of being punished from the average actual risk, although the variation is hard to measure. Studies indicated a moderate or weak relationship between perceived and actual risk of offenders (e.g., Kleck et al., 2005), suggesting that perceived risk is always influenced by other factors. In the current theme, individual characteristics and crime milieu characteristics contribute to the variation on drug offenders' perception of risk and use of strategy.

##### Individual Characteristics

People with specific characteristics become flexible in perceiving risks, acting out planned strategies, and avoiding punishment. Examples illustrate that gender (Jacobs and Miller, 1998), age (Paternoster, 1989) and social attachment (Ekland-Olson et al., 1984; DeJong, 1997) affect the action of punishment avoidance.

In most criminal subcultures, gender inequality exists (Jacobs and Miller, 1998). Women are more likely to perceive risks than men because they have lower fault tolerance in society (Carbone-Lopez, 2015). Women prefer not to implement the detection avoidance strategies that men frequently use, even if they share similar motivations. Jacobs and Miller (1998) identified that female drug dealers developed female-oriented restrictive deterrence strategies that exploited gender and normative beliefs about femininity to render an antagonistic audience neutral or perhaps even friendly.

In addition to gender, age also affects how drug offenders perceive risk and adopt strategies. Adolescents who are potential marijuana users perceive a higher perception of risk as they age; in other words, they become sensitive to a set of opportunities to commit delinquency (Paternoster, 1989).

As for the social attachment, drug offenders with solid bonds with conventional society (marriage and employment) are likely to reconsider risk before the crime. They fear losing the investment they have made in prosocial domains, leading them to commit crimes less frequently and a longer time before re-arrests (DeJong, 1997). Compared to conventional social bonds, ties with other offenders also variate the drug dealers' risk perception. Drug dealers in a dense and closed criminal network perceive less risk as they trust their co-actors; therefore, they discourage the formation and maintenance of weak ties and act as a restrictive deterrence strategy (Ekland-Olson et al., 1984).

Psychological status is another relevant individual characteristic that affects drug offenders' perception of risk and subsequent behavior. Drug offenders with experience in avoiding detection undermine risk sensitivity, as they reckon that they are more capable of escaping detection than anyone else and they become overly confident and reckless when carrying out crimes (Piliavin et al., 1986; Jacobs, 2010; Carbone-Lopez, 2015). Correspondingly, offenders who have been previously deterred are likely to produce a “reset” estimation (Pogarsky and Piquero, 2003) since they believe that arrest is rare and unlikely to occur again so soon afterward (Gallupe et al., 2011; Dickinson and Wright, 2015).

In addition to the experience in avoidance or being deterred, the existence of co-offenders also spurs drug offenders' self-serving bias and compromises their perception of risk. Accomplices decrease the fear of detection and generate social support for severe illegal acts. Co-offenders in drug offenses offer a greater feeling of control, scatter blame for the crime, and foster feelings of invulnerability; thus, the perception of risk is further compromised and spurs on individual participants (Johnson and Natarajan, 1995; Jacobs and Miller, 1998; Cross, 2000).

Another psychological status that impairs drug offenders' perception of risk is the perceived benefits of crime. The longer the drug offenders make a “career” in drug crime, the more immersed they become in the lucrative lifestyle, which reduces their perceptions of risk and boosts the perceptual rewards of crime (Ekland-Olson et al., 1984; Jacobs, 1993; Moloney et al., 2015). However, drug cultivators are an exception; they are involved in considerable planning and investment. Starting a cultivation site, large or small, can take several months with ongoing maintenance and care (Nguyen et al., 2015). Thus, drug cultivators have to remain sober and cautious of risk changes to readily adjust the drug plants, such as reducing the area cultivated.

##### Crime Milieu Characteristics

Recent research has focused on how crime milieu affects offenders' perceptions and responses to risk (Pratt et al., 2006; Piquero et al., 2011). The crime milieu is full of unexpected and twisted events which spur offenders' fast response. Offenders with a present-minded propensity are more responsive to unexpected risks and have a greater capacity to adapt to them when compared to those with a future-minded propensity (Jacobs and Cherbonneau, 2018). Being present-minded assists offenders in committing crime successfully where rationality is strictly limited. It is consistent with the concept of “mindfulness” in psychology, which stresses a capacity that helps decision-makers to block out the “noise” that hinders effective choices in unpredictable settings (Jacobs and Cherbonneau, 2018).

Drug dealing is a socially situated phenomenon (Dickinson and Wright, 2015). Drug offenders have to pay attention to the crime milieu in which the crime is about to be committed. For example, when considering natural surveillance, dealers prefer to adopt immediate transactions in a commercial area with a high level of both vehicle and pedestrian traffic. When considering formal surveillance, they prefer to avoid places with CCTV (Piza and Sytsma, 2016). Sometimes, the crime milieu is full of complexity. Drug offenders have to deal with multiple risks at the same time. It has been revealed that drug dealers have to deal on busy street segments even if there are intensified police patrols or CCTV cameras since the buyers often show up there. They develop detection avoidance strategies, including walking around and not staying in a single spot for long periods, hiding drugs in off-person stash spots, and being careful to keep their faces or bodies out of reach of the view of CCTV cameras (Bernasco and Jacques, 2015).

#### Skills in Crime

Crime skills work as a guidance for offenders to implement restrictive deterrence strategies. It lets offenders know how effective their efforts are and helps them adjust strategies in real-time (Topalli et al., 2015). Two ways that offenders acquire their skills in crime to enhance their performance are self-reflection and collective wisdom.

##### Self-Reflection

Drug offenders' crime skills largely depend on the experiential learning effect; in short, offenders learn by doing (Gallupe et al., 2011). Regardless of an experience of failure or success, experience is always a chance to advance an evolving crime-specific learning curve. Even spending time in jail stimulates restrictive deterrence. It has been found that imprisonment is related to an increased likelihood of ongoing violation for certain criminals (DeJong, 1997). Drug offenders who have long intervals before re-arrests have learned from earlier failures and have personally enacted restrictive deterrence strategies (Gallupe et al., 2011). Learning from personal experience enables drug offenders to survive longer and hence expand the scale of their operations.

The acquisition of crime skills through self-reflection is subtle. Many offenders do not notice the improvements so they deem criminal skills a certain intuition or instinct instead of an intellectual process (Johnson and Natarajan, 1995; VanNostrand and Tewksbury, 1999). It is undeniable that the more proficient the offenders in committing crime, the more natural the crime skill becomes (Nee and Ward, 2015), but it does not obscure the fact that crime skill is built up through learning. Like other specialzed criminals, drug offenders have to devote time and energy to integrate in specific scenes to acknowledge the social nuances within drug markets. Instead of intuition, repeated exposure in observing the streets enables drug offenders to identify undercover police officers by their movements, speech, and actions (Jacobs, 1996a; VanNostrand and Tewksbury, 1999; Jacques and Reynald, 2012). In some cases, nuanced changes in the accumulation of crime skills facilitate restrictive deterrence. For instance, Gallupe et al. (2011) suggested that punishment avoidance techniques can be more successful if the drug offenders conduct well-thought-out adjustments rather than impulsively implementing a complete revamp.

##### Collective Wisdom

Collective wisdom is more important for facilitating the learning process than self-reflection, especially for novices. Novices have limited experience in recognizing undercover police officers. They need vicarious experience to form punishment avoidance strategies. Re-offenders also rely on vicarious experience, as criminal experience is not only obtained based on how many times a crime is committed but also by how many types of crime are committed (Knowles, 1999). Under the screening of collective wisdom, useless punishment avoidance strategies are discarded and effective ones are pursued. Gossip plays an indispensable role in spreading the collective wisdom among active drug dealers (VanNostrand and Tewksbury, 1999; Dickinson and Wright, 2015). Drug dealers maintain informal information channels to keep track of police routines, such as shifts or patrol timetables (Jacobs, 1993; Johnson and Natarajan, 1995). They keep an eye on and gossip about clients, staff, associates, and suppliers who have had some contact with police or have recently behaved dubiously (Dickinson and Wright, 2015). Besides verbal communication, observing others' dealing activities is essential to understand the local drug markets, such as nuanced details while trading (Johnson and Natarajan, 1995; Jacobs, 1996a).

Learning from vicarious experience, drug offenders accelerate their learning curve (Bouchard and Nguyen, 2010; Fader, 2016a; Malm et al., 2017); however, not all drug offenders take advantage of collective wisdom. Some offenders proactively or passively obtain less access to drug organizations and information (Ekland-Olson et al., 1984; Jacobs and Miller, 1998; Erickson et al., 2013; Moloney et al., 2015). Besides, offenders do not blindly obey every instruction that the collective wisdom provides. Reactions to the gossip rely on how gossip subjects are caught, the social distance between listeners, and gossip subjects and sources (Dickinson and Wright, 2015). To illustrate, when hearing gossip about possible police informants, drug dealers commonly prevent connections with all or any of associates considered as police informants, at least for a short time. However, they would not alienate a recent associate who was arrested, if it was for a traffic matter. Additionally, they prefer to avoid a gossip subject when they are close to the gossip source and are less likely to avoid gossip when they are close to the gossip subject.

## Discussion

Conducting a meta-synthesis of the findings from 34 studies, this systematic review offers evidence relating to drug offenders' restrictive deterrence. Two prominent themes, namely “restrictive deterrence strategy” and “deterrability and restrictive deterrence,” emerge as a picture that depicts the whole process of drug offenders' restrictive deterrence and fit well in the certainty–severity framework of punishment (Figure 2).

Perhaps the most important conclusion of this review relates to the finding that the two types of restrictive deterrence strategies are explored equally in the reviewed papers. Restrictive deterrence strategies directly influence “whether” (certainty reduction) and “how” (severity mitigation) drug offenders will be punished. The parity of discussion between the severity and the certainty of punishment is uncommon in prior deterrence studies. The imbalanced topic distribution, specifically that most of the studies focused on the certainty of punishment, may be due to the different deterrent effects of certainty and severity of punishment. Firstly, it is generally accepted that the certainty of punishment exerts a significantly stronger and more stable deterrent effect on offenders than the severity of the punishment (DeJong, 1997; Pogarsky, 2002; Nagin and Pogarsky, 2003). Secondly, the deterrent effect of the severity of punishment relies on the certainty of punishment. As Beccaria (1963, p 58) wrote, “[t]he certainty of a punishment, even if it be moderate will always make a stronger impression than the fear of another which is more terrible but combined with the hope of impunity; even the least evils, when they are certain, always terrify men's minds”.

Although the severity of punishment has been devalued compared to the certainty of punishment, it acts as a significant catalyst that stimulates the whole deterrence process. Roche et al. (2020) revealed that offenders' perception of punishment severity significantly affected certainty. Furthermore, evidence of the anchoring effect from behavioral economics indicated that an individual is influenced by a specific number (or “anchor”) when making a statistical estimation (e.g., about a probability), and unintentionally keeps the statistical estimation close to the anchor (Tversky and Kahneman, 1974). Studies revealed that an individual's perceived certainty of risk is highly volatile and one must rank certainty by anchoring the reality, and the anchor here refers to the perceived severity of consequences for committing different offenses (Nagin, 1998; Pogarsky et al., 2018; Thomas et al., 2018).

Under the terrain of restrictive deterrence, drug offenders are no less apprehensive about the severity of punishment than its certainty. This could be due to the strategy of severity mitigation, which influences the extent of using the strategy of certainty reduction. To illustrate, a marijuana seller is less concerned about the timing and location of sales than a heroin dealer. A drug dealer with 0.01 grams of heroin on their person is less likely to care if they dress or behave in a way that will attract the attention of the police than a dealer with 100 grams of heroin. Theoretically, this echoes the aforementioned idea that the perception of the severity of punishment sets an anchor for that of certainty (Nagin, 1998; Pogarsky et al., 2018; Thomas et al., 2018). Another potential explanation is that, among deterrable offenders, the severity of punishment provides a more significant deterrent effect than the certainty of punishment (Pogarsky, 2002). In this way, the severity effect reasserts its vital power throughout the deterrence process.

It is worth noting that the severity of punishment is primarily examined in quantitative studies and certainty of punishment is usually discussed in qualitative studies. These two observations may reveal the difference in design between qualitative and quantitative studies. Qualitative studies, using mainly semi-structured interviews, usually design interviews with a relatively broad range of questions and do not strictly separate the severity of punishment from the certainty of punishment. In conjunction with what has been mentioned earlier that the certainty of punishment has a higher profile in deterrence research overall, it is easy to attribute the role, effect, or importance of severity of punishment to the certainty of punishment when interpreting drug offenders' responses to punishment. In contrast, quantitative research can clearly separate the two elements through questionnaire design and examine and demonstrate the role of severity of punishment while controlling for the role of certainty of punishment. The severity of punishment can also be explored interactively with other variables, which facilitates the identification of the role of severity of punishment in a given population or a given situation and tap into its once-overlooked position. Therefore, the qualitative study tends to examine the role of certainty of punishment, while the quantitative study is better equipped to uncover the role of severity of punishment.

The second key conclusion from this review is that the perception of risk is not only an inhibitor in using restrictive deterrence strategies, but also a facilitation of strategy differentiation. First, we found that due to the psychology of self-serving bias, drug offenders with less perception of risk implement restrictive deterrence strategies with confidence. This is consistent with the literature on the perceptual risk, which states that the lower the perception of risk, the more significant the crime (e.g., Pratt et al., 2006). However, we also discovered that the perception of risk motivates, rather than undermines, drug offenders to innovate strategies for committing crimes. For instance, female drug dealers struggled in a male-dominated field to innovate new strategies to avoid detection. Drug dealers selling drugs on CCTV-equipped streets develop a strategy to move without having their faces be captured by a camera. Such a contribution of risk perception to a cautious mindset is consistent with “flaw hunting” (Walsh, 1986), which refers to the notion that offenders sometimes utilize the high perception of risk of getting caught as an incentive for proper planning (Cherbonneau and Copes, 2006; Jacobs and Cherbonneau, 2014, 2016).

The final conclusion of this review is that the formation of crime skill relies on a combination of the slow internalization of self-reflection and the rapid input of collective wisdom. Self-reflection leads to nuanced adjustments of restrictive deterrence strategies. At the same time, collective wisdom accelerates the progress of skill learning because it reduces the individual's cost of trial and error. Perhaps because of the two different contributions to the speed and magnitude of crime skill learning, crime skill formed through self-reflection is not considered a learning process and is thus classified as an intuition. This echoes the findings of offenders' Bayesian learning based on personal experience. Bayesian learning is a way in which individuals incorporate newly learned information to update subjective prior beliefs. Anwar and Loughran (2011) found that the weight of unobserved signals (including peers' experience) when offenders consider potential risk is nearly eight times greater than the weight of considering their own arrest rate. The slight weight that is put on personal experience implies that this component is not being taken seriously.

While the reviewed research revealed fruitful restrictive deterrence strategies and their potential prerequisites, it still leaves room to explore uncharted topics that can promote our understanding of the issues. First, restrictive deterrence strategies were mainly discussed in the pre-arrest context, and future research could further explore strategies used during and after arrest. Before the arrest, by the use of proactive situational control over the context, offenders can minimize their chances of being arrested. However, this much focuses on the context of pre-arrest, delivers an incorrect presumption that drug offenders do not respond to risk once they are arrested. An arrest does not equate with the final sentence, e.g., imprisonment. Punishment is a system of conditional probabilities: restrictive deterrence strategies before an arrest can influence the outcome of punishment, and restrictive deterrence strategies after an arrest can achieve this effect as well. Between arrest and the final sentence, a series of judicial proceedings can affect the outcome, such as prosecution, conviction, and the dismissal of charges. As the “Cooperating with police” section of the current review shows, drug offenders beg or cooperate with the police to mitigate the expected severity of punishment. It is an evidence of the restrictive deterrence strategy adopted during and after arrested. Similar restrictive deterrence strategy has also been found in the study of other offenders. Sex worker, for example, might be very polite and compliant with police during arrest in the hope that they would be charged with a less severe crime (Dewey and Germain, 2014). In addition, offenders make decision on guilty plea or withdraw it in the hope that the punishment would be changed (Cheng et al., 2018). It is evident that offenders have to negotiate and deal with the authorities during and after arrest. They may come up with a completely different restrictive deterrence strategy than that used pre-arrest. Therefore, exploring the after-arrest strategy provides insights into how offenders negotiate with authority.

Second, determining the facilitative effect of the perception of risk on the innovation of restrictive deterrence strategies is an area for future research. While much of the broader deterrence literature has quantified the role of the perception of risk in curbing crime or an individual's intention to commit a crime (e.g., Pratt et al., 2006), in ethnographic studies of restrictive deterrence it is implied that the perception of risk stimulates innovation in crime strategies (e.g., Jacobs and Miller, 1998). This is a relatively novel idea that emphasizes the vital power of the perception of risk from the opposite perspective. Nevertheless, it is unclear what type of perception of risk motivates offenders' innovation or planning ability and willingness rather than acting as a deterrent. For instance, increased offender risk perception and vigilance may be an early warning signal. Indeed, prior restrictive deterrence research based on a sample of hackers suggested that a warning banner significantly reduces the duration of trespassing incidents (Maimon et al., 2014). The mediation that connects the perception of risk and strategy innovation is also unclear, both psychological and social. Prior research revealed that offenders with better emotional management are good at translating perceptions of risk into better risk coping strategies, and peer support reinforces their emotional management (Jacobs and Cherbonneau, 2017). We definitely do not wish to only dwell on how the perception of risk exerts its facilitative effect on strategy differentiation. Exploring the aforementioned issues would allow the literature to better understand how the two roles of perception of risk (curbing crime/facilitating strategy development) reconcile.

Another future research line for exploration is to look into the impact of collective wisdom on crime skill learning and strategy use. The influence of collective wisdom on offenders' crime skills is more significant than individual reflection regarding speed and quantity. However, the effectiveness of collective wisdom highly depends on the offender's closeness to other offenders or crime organizations. It is noted that the connection to criminal groups is beneficial for drug offenders to obtain advanced and effective strategies (Ekland-Olson et al., 1984; Jacobs and Miller, 1998; Moloney et al., 2015). Others have attempted to quantify the role of collective wisdom (peer experience) in the learning process (e.g., Pogarsky et al., 2004), but as of yet it remains unclear how ties play into this. Hence, research can be extended to explore what kind of tie is efficient to diffuse collective wisdom on crime skill and through what kind of communication paths offenders are more likely to accept and adopt the crime skill. In other words, how relationships affect the rate of transmission and acceptance of collective wisdom. Studying different channels that spread crime skills and making comparisons among them can generate insights about the iteration of restrictive deterrence. Furthermore, the literature needs more details on restrictive deterrence advances in drug offender groups.

Interpretation of our results should be tempered by several limitations. First, although the literature search was comprehensive, only a small number of studies could be included compared to previous literature reviews focusing on deterrence or drug criminality (e.g., Pratt et al., 2006). Simply put, only studies that considered and discussed restrictive deterrence of drug offenders seriously were included. Studies that merely referred to crime strategies of drug offenders in a broader research question, e.g., drug economy (e.g., Dickinson, 2020), were excluded but may provide additional insights. Second, we specifically selected only English studies when conducting data search, which means that studies published in other languages may have reported different conclusions and impacts. Therefore, this review is limited in its evaluation of cross-cultural aspects in restrictive deterrence of drug offenders. Finally, several important questions remain unanswered in the current review. For instance, which type of deterrent strategies is most effective against which kind of drug offenses (e.g., using, selling, cultivating drugs, etc.), and which type of restrictive deterrence strategy works best in which kind of situations (e.g., gender, drug type, time, places). We were not able to perform further analysis due to a general dearth of quantitative study in the literature related to restrictive deterrence of drug offenders.

## Data Availability Statement

The original contributions presented in the study are included in the article/Supplementary Material, further inquiries can be directed to the corresponding author.

## Author Contributions

XG organized the database and wrote the first draft of the manuscript. TWL contributed to reviewing and editing the manuscript. Both authors contributed to manuscript revision, read, and approved the submitted version.

## Conflict of Interest

The authors declare that the research was conducted in the absence of any commercial or financial relationships that could be construed as a potential conflict of interest.

## Publisher's Note

All claims expressed in this article are solely those of the authors and do not necessarily represent those of their affiliated organizations, or those of the publisher, the editors and the reviewers. Any product that may be evaluated in this article, or claim that may be made by its manufacturer, is not guaranteed or endorsed by the publisher.
