# Supplementary material for: Restrictive Deterrence in Drug Offenses: A Systematic Review and Meta-Synthesis of Mixed Studies
Source: Front Psychol. 2021 Aug 25;12:727142. doi: 10.3389/fpsyg.2021.727142 (PMC8423896; doi:10.3389/fpsyg.2021.727142)
Supplement: Supplementary file 1 [file Data_Sheet_1.docx]

# Appendix 1. Search Strategy

(“drug offender*” OR “drug criminal*” OR “drug dealer*” OR “drug seller*” OR “drug trafficker*” OR “drug deliverym*” OR “drug importer*” OR “drug supplier*” OR “drug broker*” OR “drug courier*” OR “drug smuggler*” OR “drug wholesaler*” OR “drug distributor*” OR “drug user*” OR “drug abuser*” OR “drug cultivator*” OR “drug producer*” OR “drug charge*”) AND (“restrictive deterren*” OR “partial deterren*” OR “apprehension avoidance” OR “arrest avoidance” OR “sanction avoidance” OR “risk avoidance” OR “punishment avoidance” OR “detection avoidance” OR “avoid arrest” OR “avoid apprehension” OR “avoid sanction” OR “avoid detection” OR “avoid police” OR “avoid risk” OR “avoid punishment”) AND (“risk perception” OR “decision making” OR “rational choice” OR “risk management” OR “deterrence theory” OR strateg* OR respon* OR surviv* OR tactics OR experience OR experiences OR experienced OR perception OR perceptions OR perceive OR perceived OR attitude OR attitudes OR belief OR beliefs OR perspective OR perspectives OR opinion OR opinions OR concept OR concepts OR thought OR thoughts OR intuition OR awareness OR comprehension OR understanding) NOT abstract(HIV or health) NOT title(HIV or health)

# Appendix 2. Descriptive information on studies in the systematic review

| **Appendix 2** Descriptive information on studies in the systematic review. | Type of offense | Drug dealers (poly-drug) | Drug users | Drug users | Drug dealers (heroin) | Drug dealers (crack) | Drug dealers (crack) | Drug dealers (crack) | Drug offenders | Drug dealers (crack) |
| --- | --- | --- | --- | --- | --- | --- | --- | --- | --- | --- |
|  | Settings | Null | Chicago, Jersey City, Oakland and Philadelphia | A mid-sized south eastern city | Western city | New York City | A medium-sized midwestern city | A midwestern city | New York City | St. Louis, Missouri |
|  | Methods | Ethnography and interviews with active dealers | Three-wave questionnaire with drug users and other deviant populations; confirmatory factor analysis; structural model | Three-wave self-reported questionnaire; logistic regression | Semi-structured interviews | Ethnography and semi-structured interviews | Semi-structured interviews | Semi-structured interviews | Survival analyzes based on lognormal model with court files | Semi-structured interviews |
|  | Sample demographics | Null | 974 drug users: 779 men, 195 women; mean age 27.8; 759 Black, 87 Hispanic, 128 White or other | Sophomore to Senior year high school students | All male; average age 48; 15 White, 14 Hispanic, 2 Asian and 1 Black | Null | 36 male, 4 female; average age 23; all Black | 36 male, 4 female; average age 23; all Black | All male; mean age 26.86–28.54; 55.7%–56.8% Black, 31.8%–36.3% Hispanic, 8.1%–11.5% White | All female |
|  | Sample size | 34 | 3300 | 1250 | 32 | 300 | 40 | 40 | 5598 | 25 |
|  | Objective | Determine how perceptions of sanctions organize interpersonal relationships and restrict the activity of drug dealing | To explore the effect of formal sanctions on various criminal behavior, including a sample of drug users | To explore the factors that affect four common delinquent offenses, including marijuana use | To explore how drug dealers unveil undercover agents | To explore how drug dealers construct their routine activities to adapt to police tactics | To explore how drug dealers identify if the trade is a buy–bust operation | To explore how drug dealers avoid detection and arrest | To explore the factors that affect the timing and probability of recidivism, including a sample of drug offenders | To explore how female drug dealers manage risk and avoid arrest |
|  | Date | 1984 | 1986 | 1989 | 1993 | 1995 | 1996 | 1996 | 1997 | 1998 |
|  | Authors | Ekland-Olson, Lieb and Zurcher | Piliavi, Gartner, Thornton and Matsueda | Paternoster | Jacobs | Johnson and Natarajan | Jacobs(a) | Jacobs(b) | DeJong | Jacobs and Miller |

| **Appendix 2** (continued) | Type of offense | Drug dealers (crack) | Drug dealers (poly-drug) | Drug dealers (crack) | Drug users (crack) | Drug cultivators (cannabis) | Drug offenders | Drug dealers (poly-drug) | Drug users | Drug dealers (crack) |
| --- | --- | --- | --- | --- | --- | --- | --- | --- | --- | --- |
|  | Settings | Honolulu | Mid-western city | New York City | Brazil | A rural region in Quebec, Canada | South Australia | Suburban, Atlanta; poor community, St. Louis | Canada | Easton, UK |
|  | Methods | Semi-structured interviews | Semi-structured interviews with drug court participants | Secondary analysis of 132 interview segments in previous project | Semi-structured interviews with long-term users | Self-reported questionnaire; Nested logistic regression models and CHAID analysis | Survival analyzes based on Cox proportional hazard models with court files | Semi-structure interviews with active street dealers | Questionnaire; semi-structure interviews with drug users | Interviews with female crack cocaine dealers |
|  | Sample demographics | 3 male, 2 female; 2 Black, 2 Caucasian, 1 Filipino | 16 male; average age 31; 80% Black, 20% White | Null | 20 male, 8 female; mean age 32 | 117 male, 58 female; mean age 15.7 | Null | age range 18–23; 25 middle class and 25 low-level drug dealers | 80 male, 42 female; mean age 35.9 | 8 female; age range 20–40; 5 African Caribbean, 2 mixed race, 1 Gypsy. |
|  | Sample size | 5 | 20 | 132 | 28 | 175 | 26819 | 50 | 122 | 8 |
|  | Objective | To explore how drug dealers counter law enforcement effort and the criminal justice system | To explore why and how drug dealers avoid detection | To explore how drug dealers manage and avoid detection | To explore how drug users deal with risks | To explore the influence of a criminal network and co-offending measures on the likelihood of arrest of juvenile offenders | To explore the influence of drug offenders’ post-arrest behavioral changes on time to re-arrest | To explore situational strategies drug dealers use to evade adversaries | To explore the effect of legal knowledge on drug user patterns | To explore the strategies that women employ to deal crack |
|  | Date | 1999 | 1999 | 2000 | 2010 | 2010 | 2011 | 2012 | 2013 | 2014 |
|  | Authors | Knowles | Vannostrand and Tewksbury | Cross | Riberio | Bouchard and Nguyen | Gallupe, Bouchard and Caulkins | Jacques and Reynald | Erickson, Van Der Maas and Hathaway | Fleetwood |

| **Appendix 2** (continued) | Type of offense | Drug dealers (poly-drug) | Drug dealers (poly-drug) | Drug offenders (meth) | Drug dealers (poly-drug) | Drug dealers (poly-drug) | Drug cultivator (cannabis) | Drug dealers (poly-drug) | Drug dealers (poly-drug) | Drug dealers (poly-drug) |
| --- | --- | --- | --- | --- | --- | --- | --- | --- | --- | --- |
|  | Settings | Suburban town | Amsterdam, the Netherlands | Missouri | St. Louis, Missouri | Null | 44 states, United States | Null | Philadelphia, Pennsylvania | Philadelphia, Pennsylvania |
|  | Methods | Semi-structured interviews | Systematic social observation; semi-structured interviews with active drug dealers | Semi-structured interviews | Semi-structured interviews and ethnographic observation | Semi-structured interviews | Questionnaire | Qualitative meta-synthesis | Semi-structured interviews with active and former drug dealers | Semi-structured interviews with active and former drug dealers |
|  | Sample demographics | 27 male, 2 female; age range18–23; 28 White, 1 Mixed | 48 male, 2 female; mean age 38.7 | All female; average age 32; all White | 29 male; average age 26.8; 27 white, 3 Black, 2 Latino; 1 Chinese-American | 120 male, 133 female; average age 21.7; 53% Black, 26% Latino, 11% Asian, 18% Mixed | 86.5% male; mean age 31.8; 82.9% White | Null | 18 male, 2 female; mean age 24.5; all African American | 18 male, 2 female; mean age 24.5; all African American |
|  | Sample size | 29 | 50 drug dealers; 262 street segments | 38 | 33 | 253 | 337 cultivators; 338 had co-offenders | 17 articles | 20 | 20 |
|  | Objective | To explore how four types of sanctions restrict drug sales | To explore factors that affect street drug dealers’ choices about where to solicit customers and make sales. | To explore how female drug users adapt use and market behaviors based on recent law changes. | To explore how gossip facilitates drug dealers’ decision making and detection avoidance | To explore how gang-involved young adults interpret the risk of drug dealing | To explore the relationship between the threat of sanctions and the size of cultivation site and number of co-offenders | To summarize and synthesize qualitative research on restrictive deterrence, including drug offenses | To explore the how family networks facilitate cost avoidance strategies by urban drug sellers and transmission of criminal capital by these networks | To explore the interaction between perceptions of risk and drug offending strategies among modern-day drug sellers |
|  | Date | 2014 | 2015 | 2015 | 2015 | 2015 | 2015 | 2016 | 2016 | 2016 |
|  | Authors | Jacques and Allen | Bernasco | Carbone-Lopez | Dickinson and Wright | Moloney, Hunt and Joe-Laidler | Nguyen, Malmb and Bouchardc | Moeller, Copes and Hochstetler | Fader(a) | Fader(b) |

| **Appendix 2** (continued) | Type of offense | Drug dealers (poly-drug) | Drug dealers (online) | Drug cultivators (marijuana) | Drug dealers (poly-drug) | Drug producers (meth) | Drug dealers | Drug dealers (poly-drug) |
| --- | --- | --- | --- | --- | --- | --- | --- | --- |
|  | Settings | Newark, New Jersey | Silk road | The United States, Belgium, and the Netherlands | Newark, New Jersey | Null | Baltimore, MD | Philadelphia, Pennsylvania |
|  | Methods | Systematic social observation | Thematic and discourse analyzes | Questionnaire | Systematic social observation; script analysis | Qualitative content analysis | Ethnographic observation and systematic social observation | Semi-structured interviews with active and former drug dealers |
|  | Sample demographics | 176 male, 24 female; 23 White, 177 Black | Null | 88% male, 12% female; mean age 28.65 | Null | Null | CCTV footage recording drug crimes that resulted in an arrest | 18 male, 2 female; average age 24.5; all African American |
|  | Sample size | 92 individual transactions across the 62 drug events, including 200 actors | 600 online sample | 359 | 62 drug events | 10 meth producing recipes | 2,340 hours of CCTV footage | 20 |
|  | Objective | To explore the defensive actions of drug sellers within open-air retail markets | To explore the strategies drug dealers use to reduce the risk of detection and arrest | To explore the relationship between the criminal network structure and risk perception | To identify the typical conditions of each necessary step during drug dealing and explore scene factors associated with the typical conditions | To explore the strategies that methamphetamine producers have adapted to get around the regulations | To explore the micro-routines of drug dealers that evade detection | To explore drug sellers’ perceptions of risk and returns in the modern context |
|  | Date | 2016 | 2017 | 2017 | 2018 | 2018 | 2018 | 2019 |
|  | Authors | Piza | Aldridge and Askew | Malm | Sytsma | Vidal | Olaghere and Lum | Fader |
